# Supplementary material for: Artificial intelligence detects awareness of functional relation with the environment in 3 month old babies
Source: Sci Rep. 2024 Jul 6;14:15580. doi: 10.1038/s41598-024-66312-6 (PMC11227524; doi:10.1038/s41598-024-66312-6)
Supplement: Supplementary file 1 — Supplementary Information. [file 41598_2024_66312_MOESM1_ESM.pdf]

Supplementary Information

Neural Networks

In supervised learning, a neural network predicts the output data y from an input data x using $y = f(x,\theta)$. In this model, θ are the parameters of the model, and f is a large and layer-structured model. A basic layer is a non-linear function that follows a linear function. This non-linear activation function enables the modelling of complex relations in the network. In addition, to provide suitability for computer hardware and feasibility of fast evaluation of the networks (regardless of their size), linear functions and layer structure are employed. For instance, consider the following example of a layer l with an input of a, and an output of c (1):

$$c_j = g(\sum_i w_{i,j} a_i + b_j) \tag{1}$$

In this equation, b_j and $w_{i,j}$ are parameters of the model, i, j are indices employed to distinguish individual nodes in layer l and layer $l+1$, respectively, and g represents the activation function. Moreover, $c_j s$ are outputs (*i.e.*, activations of the node j) and are later employed in a similar manner to calculate the activations of the layer $l+2$. The activation c_j can be assumed an unnormalized probability for a feature specific to the node j. In a layer, nodes are generally arranged in a 3D tensor. However, if the input is inherently 2D (*e.g.*, an image), the following layers will have two dimensions in accordance with the image dimensions. In this paper, they are referred to as spatial or image dimensions, while the remaining layers are referred to as channel dimensions.

b_j and $w_{i,j}$ are learned through the evaluation of the model on the test data set $(\bar{y},\bar{x})$ and through the minimization of the error $l(\bar{y}, f(\bar{x},\theta),\theta)$ for θ. In this error, l is a loss function that describes the distance between $\bar{y}$ and $f(\bar{x},\theta)$ on θ similar to prior instances. In almost all cases, a Stochastic Gradient Descent (SGD)-based optimizer is employed. Often, a layer's input can be assumed shift-invariant (*e.g.*, for image inputs). In such cases, the utilization of convolutional layers seems logical, where the weights are shared so that if the relationship between a_i and c_j in the spatial dimension is similar to a_l and c_k, then $w_{i,j} = w_{l,k}$ and $b_{i,j} = b_{l,k}$. In addition, if a_i is spatially far from c_j, then it can be assumed that $w_{i,j} = 0$, thus limiting the field of view for c_j.

2D and 3D Convolutional Neural Networks (CNNs)

The function of a convolutional neural network (CNN) is to learn the mapping between input data and output data[1]. A 2D-CNN processes input images by first applying a sequence of matrix multiplications called kernel filters with a sample size (P_i, Q_i) in the ith layer and then summing the results. Identifying the most important features in that image is the objective[2]. The output V_{ij}^{ab} centred at (a,b) for the jth feature map and the ith layer can be stated in (2).

$$V_{ij}^{ab} = tanh\,(\sum_m \sum_{x=0}^{P_i-1} \sum_{y=0}^{Q_i-1} W_{ijm}^{xy}\, V_{(i-1)m}^{(a+x)(b+y)} + b_{ij}) \tag{2}$$

The non-linearity operation is tanh used on the kernel output and the biases is b_{ij}. ${W_{ij}}_m^{xy}$ is the output of kernels centered at (x,y) for the kth feature map. Also, m is the indexing parameter over a feature maps set connected to the current feature map in the $(i-1)^{th}$ layer. The output of a convolutional layer is typically a feature map that is reflective of the learned features from the input image. Therefore, when R_i represents the third dimension of the kernels, the output centered at (a,b,c) for the jth feature map and the ith layer is formulated in 3D-CNN (3). The kernel output is ${W_{ij}}_m^{xyz}$ centred at (x,y,z) for the kth feature map. Also, m represents feature maps indexes.

$$V_{ij}^{abc} = tanh\,(\sum_m \sum_{x=0}^{P_i-1} \sum_{y=0}^{Q_i-1} \sum_{z=0}^{R_i-1} W_{ijm}^{xyz}\ V_{(i-1)m}^{(a+x)(b+y)(c+z)} + b_{ij}) \tag{3}$$

Capsule Neural Network (CapsNet)

It has been suggested that the novel artificial neural network known as CapsNet can approach the biological representation of the human brain in terms of its hierarchical connections in a more precise way[3]. Modules, represented as capsules in the brain, are highly proficient at controlling different visual stimuli and encoding information[4]. Pooling, which is usually employed for dimension reduction in CNN networks, does not exhibit this characteristic. Each capsule in a CapsNet layer forward propagates

data to the capsule in the layer above it, resulting in a hierarchical structure. Table 1 displays a comparison between CapsNets and neural networks.

Traditional neural networks have an output equal to the sum of the weighted neurons a_j computed in the last layer. The output h_j, which is scalar, is calculated with a nonlinear activation function. However, in CapsNet, instead of treating each neuron individually, a capsule treats the entire group of neurons as a whole. To do so, it uses vector capsules instead of scalar output feature indicators (activation vectors u_i). The activation vectors with weighted coefficients are multiplied by a matrix W_{ij}. Then, the vectors s_j in the preceding layer are weighted. Finally, the vector is scaled between zero and unit length using summing and a squashing function (v_j).

		CapsNet	Traditional neural networks
Input from previous capsule/neuron		Vector (u_i)	Scalar (x_i)
Operation	Affine Transform	$\hat{u}_{j\vert i} = W_{ij}u_i$	-
	Weighting Sum	$s_j = \sum_t c_{ij}\, \hat{u}_{j\vert i}$	$a_j = \sum_i w_i\, x_i + b$
	Nonlinear activation	$v_j = \frac{\lVert s_j \rVert^2}{1+\lVert s_j \rVert^2} \frac{s_j}{\lVert s_j \rVert}$	$h_j = f(a_j)$
Output		Vector (v_j)	Scalar (h_j)

Table 1. A comparison between traditional neural networks and CapsNet

Figure 1 displays the layers that comprise the original CapsNet model, which are the convolution layers, the primary capsule layer, the DigitCaps, and the fully connected layer. The model receives a handwritten image of digits. Conv1 and Conv2 are two Convolution layers with the same kernels and different strides. In these Convolution layers, the Rectified Linear Unit (ReLU) is used as the nonlinearity. Therefore, the feature maps that are generated by Conv1 and Conv2 are different. By modifying the feature maps, the primary capsule layer is created. It functions as the Capsule's input layer, and its purpose is to construct the corresponding vector structure. The output of this layer's reshaping is used as a vector input in the subsequent layer. DigitCaps are then used to determine the loss function for the classification objective (encoder component), while the fully connected layers used for image reconstruction (decoder part) serve as network regularisations that avoid overfitting. Between primary capsules and DigitCaps, the DR method is used to update the necessary calculations and parameters between full connections. As for updating the parameters, CapsNets use both DR and the traditional back-propagation technique[3].

Methods

In the preceding sections, we discussed the Deep Learning methods used in the paper (including FCNet, 1D-Conv, 1D-CapsNet, 2D-Conv, and 2D-CapsNet). Detailed model specifications, including parameter settings, layer configurations, and filter choices, are also provided for a comprehensive understanding.

Fully connected networks

FCNets are used as a generic framework for some inputs such as text and extracted features because of their robustness. Therefore, a 1D vector of the histogram-based features was fed to our four-layer FCNet architecture when reducing the layer sizes (Figure 2). Also, to avoid overfitting, our network was formed so that a dropout layer followed each fully connected layer. The $SoftMax$ layer was connected to the last fully connected layer with $Fc = 5$ for the classification of the five different stages (cf. Figure 8 in the paper).Two-hundred and forty filters were used for the first convolution and $Drop\ out = 0.5$ was chosen after optimization.

Convolutional neural networks

The input of our proposed 1D-Conv network with $kernel\ size = 3$, $max\ pooling = 3$, and $stride = 3$ is a 1D vector of the histogram-based features. To reduce the negative effect of overfitting, the output of the last dropout layer was flattened into a 1D vector, and the fully connected layer reduced the dimensionality using a max pooling before feeding into a $SoftMax$ layer for classification. Figure 3 demonstrates the architecture of the proposed 1D-Conv. Sixteen and 32 filters were chosen for the first and second convolution, respectively, after optimization.

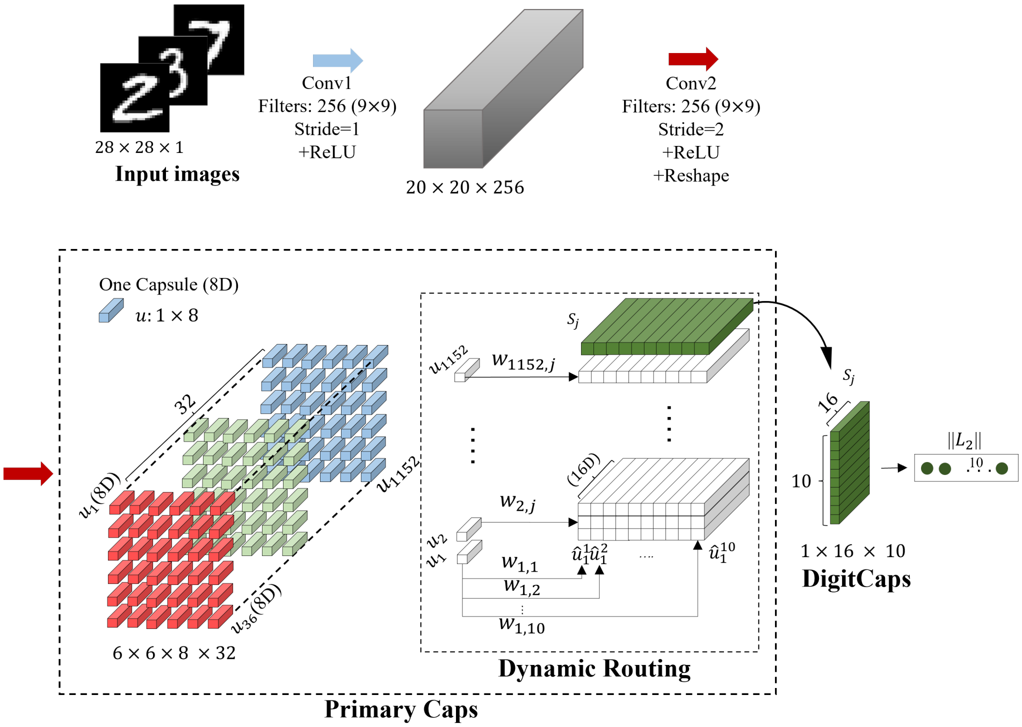

Figure 1. Structure of the CapsNet[5]. As an input image, the model receives a handwritten digit from the MNIST database of handwritten digits [67]. For 10 distinct classes, the system learns to encode images of size 28x28x1 into a 16D vector of instantiation parameters (DigitCaps). To accomplish the extraction of local features, the convolution layers are traditional convolution layers with a rectified linear unit (ReLU) activation function. There are two layers of convolution first: conv1 uses a 9x9 convolutional kernel and ReLU with a stride of 1, second: conv2 uses a 9x9 convolutional kernel and ReLU with a stride of 2. The first conversion yields 256 20x20 feature maps, whereas the second yields 256 scalar-filled 6x6 $(32x8x6x6)$ feature maps. This result is converted to produce 32 $6x6$ maps with 8D vectors. 1152 prediction × 10 classes equals 11520 weighted matrices W_{ij}.

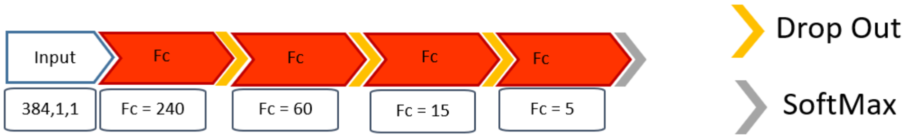

Figure 2. The proposed FCNet architecture after hyperparameter optimisation. FCNet is designed to avoid overfitting by including a dropout layer after each fully connected (Fc) layer.

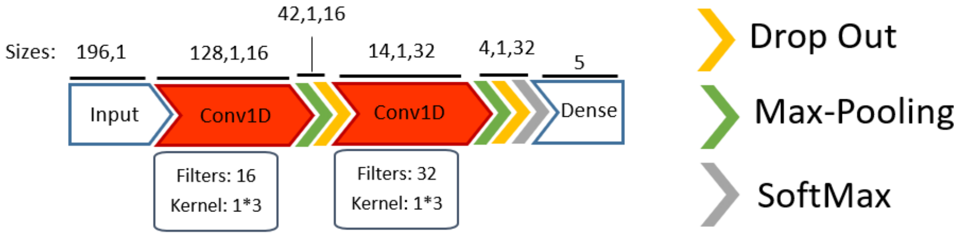

Figure 3. The proposed 1D-Conv architecture after hyperparameter optimisation. In 1D-Conv, max pooling is applied to reduce the dimensionality.

We created a long 1D vector: $hist_{combined\_1D} = [hist_{part1}, hist_{part2}, ..., hist_{partn}]$ by appending histogram features of individual body parts to generate bilateral fused features for Hands, Knees, Feet, and Full-body. To maximise the use of spatial information between different body parts during movement, we proposed an additional 2D-CNN. In the latter, a 2D matrix shape called $hist_{combined\_2D}$ is formed by reshaping the 1D feature vector to a 2D matrix. Each row of the 2D matrix contains the histogram of features derived from a single body part.

$$hist_{combined\_2D} = \begin{bmatrix} hist_{part1} \\ hist_{part2} \\ . \\ . \\ . \\ hist_{partn} \end{bmatrix}$$

As shown in Figure 4, the proposed 2D-Conv consists of two 2D convolution layers followed by a max-pooling and dropout. In this architecture, kernel size was 3 and stride was 1 for convolutions, and the output was downsampled with max-pooling using the same kernel size and $stride = 2$. The output of the final dropout layer was flattened to a 1D vector, which was then reduced in dimension by a fully connected layer before being fed into a $SoftMax$ layer for classification. Four and eight filters were chosen for first and second convolution, respectively, after optimization.

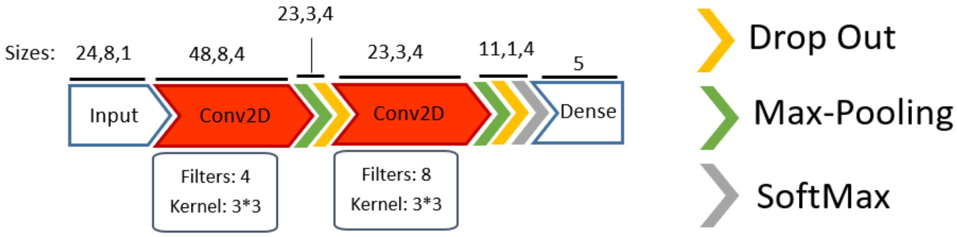

Figure 4. The proposed 2D-Conv architecture.

Algorithm 1: Training and Backpropagation

for numbers of epochs
  for iterations (batches)
    for numbers of routing (r)

$$b_{i,j}(initialized)^{(r=1)} = 0$$

$$b_{i,j} \rightarrow c_{i,j} \quad \text{; Coupling coefficient (Softmax)}$$

$$S_j{}^{(r)} = \sum_i c_{i,j} \,.\, \hat{u}_i^j{}^{(r)} \quad \text{; Weighted sum}$$

$$V_j{}^{(r)} = Squash\left(S_j{}^{(r)}\right) \quad \text{; Product vector}$$

$$= Squash\left(\sum_i c_{i,j} \,.\, \hat{u}_i^j{}^{(r)}\right)$$

Dot Product: $\hat{u}_i^j \,.\, V_j{}^{(r)}$

$$b_{i,j}{}^{Updated} \leftarrow b_{i,j} + \hat{u}_i^j \,.\, V_j{}^{(r)}$$

$$\leftarrow b_{i,j} + \hat{u}_i^j{}^T \,.\, V_j{}^{(r)}$$

$$\leftarrow b_{i,j} + \hat{u}_i^j{}^T \,.\, Squash\left(\sum_i c_{i,j} \,.\, \hat{u}_i^j{}^{(r)}\right)$$

return V_j *(end for routings)*

The loss function for the correct category:

$$L_J = k.max(0, 0.9 - \|V_j\|)$$

Backpropagation starts:

Weighted matrices: $w_{i,j}{}^{updated} \leftarrow w_{i,j}$

Convolution layers: $filters^{updated} \leftarrow filters$

  return (end for iterations)
return (end for epochs)

Figure 5. Routing by Agreement Algorithm[3].

Capsule Neural Networks

To prevent losing information when using pooling in CNNs, we leveraged the CapsNet architecture, which utilises a novel technique called Dynamic Routing (DR) to establish an optimal path between different capsule layers (**Algorithm.1 in Figure** 5). This enables the construction of hierarchical relationships between features while requiring fewer training samples. Also, CNNs ignore the positional correlation among local features in the spatial domain due to their focus on translation invariance and parameter sharing for efficient and robust representation learning.

To mitigate these issues, we used the same convolution layer settings for 1D and 2D CNNs, then removed the max-pooling before forming features and encapsulating them in Primary Capsules (PCs). Dynamic routing was then applied to send the most relevant primary capsule to the class capsule. Figures 6-7 display the proposed CapsNet architectures, which can receive both 1D and 2D histograms as input. As stated in results, we can either generate a 1D-CapsNet or a 2D-CapsNet, depending on the input dimension. After parameter optimization, $Kernel\ filter = 3$ was used for both CapsNet models, while 32 and 16 filters were chosen for the number of filters in 1D- and 2D-CapsNet, respectively, in convolution layers.

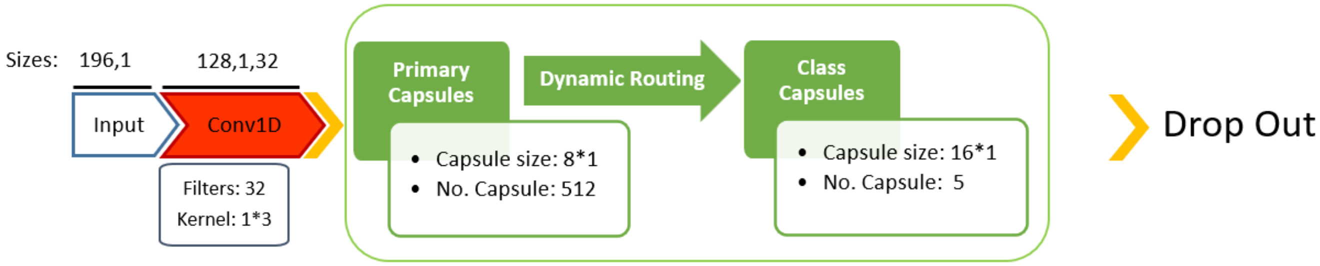

Figure 6. The proposed 1D-CapsNet architecture after hyperparameter optimisation.

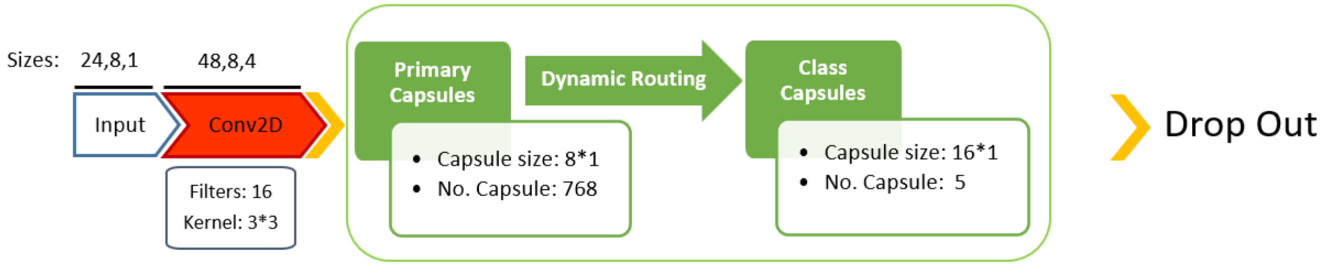

Figure 7. The proposed 2D-CapsNet architecture after hyperparameter optimisation.

Results

Hyperparameter Settings

Table 2 lists the hyperparameters optimised for each model. Since no hyperparameter optimisation was employed in previous work[6], we used a range of parameters to generalise approaches (Table 2). For FCNets, we optimised the hyperparameters using FCNet layers $= (240, 60, 15)$ and $dropout\ rates = (0.5, 0.7, 0.9)$. The first layer filters $(8, 16, 32)$ and the second layer filters $(8, 16, 32)$ for the 1D-Conv approach were also optimised. Similarly, to 1D-Conv, we chose the following first- and second-layer filters for 2D-Conv $= (4, 8, 32)$, $Kernel\ size = (3, 4, 5)$, and the number of filters $= (8, 16)$ for optimising parameters in both the 1D and 2D-CapsNet approaches.

Models	Hyper-parameter 1	Hyper-parameter 2
**FCNet**	# first FCNet layer: (240, 150, 100)	Dropout rates: (0.5, 0.7, 0.9)
**1D-Conv**	# first-layer filters: (8, 16, 32)	# second-layer filters: (8, 16, 32)
**2D-Conv**	# first-layer filters: (4, 8, 32)	#second-layer filters: (4, 8, 32)
**1D-CapsNet**	Kernel size: (3, 4, 5)	# layer filters: (16, 32)
**2D-CapsNet**	Kernel size:(3, 4, 5)	# layer filters: (8, 16)

Table 2. Hyper-parameters selection.

Confusion Matrix

Stage B2 emerges as a consistent recipient of the most accurate labels. The confusion matrix serves as a comprehensive snapshot, illustrating the model's proficiency in correctly identifying and classifying instances corresponding to Stage B2, thereby reinforcing the notion that accurate HJD detection is particularly reliable during this specific stage of analysis (Figure 8).

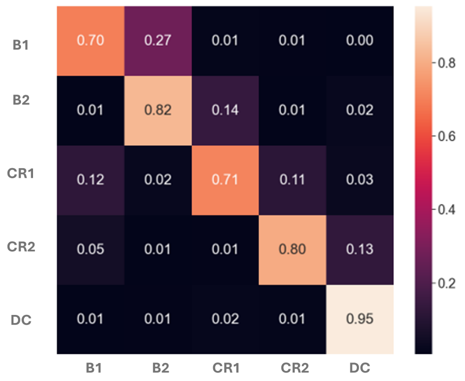

(a) Subject 1-Feet

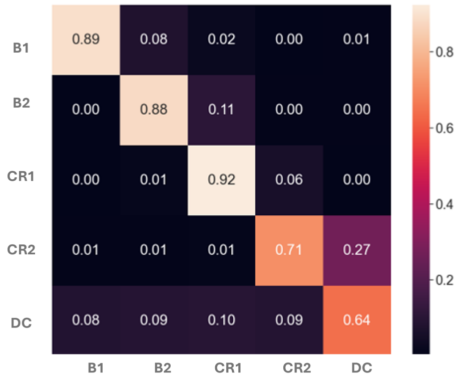

(b) Subject 2-Feet

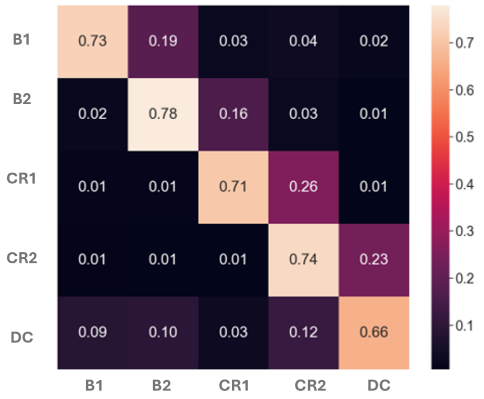

(c) Subject 3-Feet

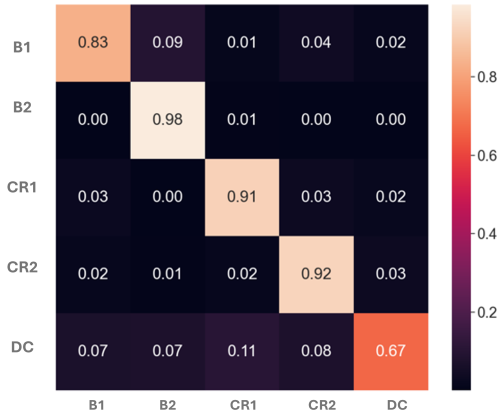

(d) Subject 4-Feet

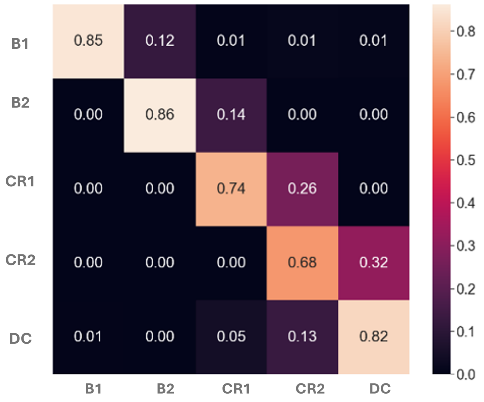

(e) Subject 5-Feet

Figure 8. The normalised confusion matrix for each subject.

References

1. LeCun, Y., Bottou, L., Bengio, Y. & Haffner, P. Gradient-based learning applied to document recognition. *Proc. IEEE* **86**, 2278–2323, DOI: 10.1109/5.726791 (1998).
2. Ji, S., Xu, W., Yang, M. & Yu, K. 3d convolutional neural networks for human action recognition. *IEEE Transactions on Pattern Analysis Mach. Intell.* **35**, 221–231, DOI: 10.1109/TPAMI.2012.59 (2013).
3. Sabour, S., Frosst, N. & Hinton, G. E. Dynamic routing between capsules. vol. 2017-Decem, 3857–3867, DOI: 10.48550/arxiv.1710.09829 (2017).
4. Hinton, G. E., Krizhevsky, A. & Wang, S. D. Transforming auto-encoders. *Lect. Notes Comput. Sci.* **6791 LNCS**, 44–51, DOI: 10.1007/978-3-642-21735-7_6 (2011).
5. Khodadadzadeh, M., Ding, X., Chaurasia, P. & Coyle, D. A hybrid capsule network for hyperspectral image classification. *IEEE J. Sel. Top. Appl. Earth Obs. Remote. Sens.* **14**, 11824–11839, DOI: 10.1109/JSTARS.2021.3126427 (2021).
6. McCay, K. D. *et al.* Abnormal infant movements classification with deep learning on pose-based features. *IEEE Access* **8**, 51582–51592, DOI: 10.1109/ACCESS.2020.2980269 (2020).
